# An experimental study of the effects of SNPs in the TATA boxes of the GRIN1, ASCL3 and NOS1 genes on interactions with the TATA-binding protein

**DOI:** 10.18699/VJGB-22-29

**Published:** 2022-05

**Authors:** E.B. Sharypova, I.A. Drachkova, I.V. Chadaeva, M.P. Ponomarenko, M.P. Savinkova

**Affiliations:** Institute of Cytology and Genetics of the Siberian Branch of the Russian Academy of Sciences, Novosibirsk, Russia; Institute of Cytology and Genetics of the Siberian Branch of the Russian Academy of Sciences, Novosibirsk, Russia; Institute of Cytology and Genetics of the Siberian Branch of the Russian Academy of Sciences, Novosibirsk, Russia; Institute of Cytology and Genetics of the Siberian Branch of the Russian Academy of Sciences, Novosibirsk, Russia; Institute of Cytology and Genetics of the Siberian Branch of the Russian Academy of Sciences, Novosibirsk, Russia

**Keywords:** GRIN1, ASCL3, NOS1, TATA-binding protein, affinity, TBP/TATA interaction, GRIN1, ASCL3, NOS1, TATA-связывающий белок, aффинность, TBP/TATA взаимодействие

## Abstract

The GRIN1, ASCL3, and NOS1 genes are associated with various phenotypes of neuropsychiatric disorders. For instance, these genes contribute to the development of schizophrenia, Alzheimer’s and Parkinson’s diseases, and epilepsy. These genes are also associated with various cancers. For example, ASCL3 is overexpressed in breast cancer, and NOS1, in ovarian cancer cell lines. Based on our findings and literature data, we had previously obtained results suggesting that the single-nucleotide polymorphisms (SNPs) that disrupt erythropoiesis are highly likely to be associated with cognitive and neuropsychiatric disorders in humans. In the present work, using SNP_TATA_Z-tester, we investigated the influence of unannotated SNPs in the TATA boxes of the promoters of the GRIN1, ASCL3, and NOS1 genes (which are involved in neuropsychiatric disorders and cancers) on the interaction of the TATA boxes with the TATA-binding protein (TBP). Double-stranded oligodeoxyribonucleotides identical to the TATA-containing promoter regions of the GRIN1, ASCL3, and NOS1 genes (reference and minor alleles) and recombinant human TBP were employed to study in vitro (by an electrophoretic mobility shift assay) kinetic characteristics of the formation of TBP–TATA complexes and their affinity. It was found, for example, that allele A of rs1402667001 in the GRIN1 promoter increases TBP–TATA affinity 1.4-fold, whereas allele C in the TATA box of the ASCL3 promoter decreases the affinity 1.4-fold. The lifetime of the complexes in both cases decreased by ~20 % due to changes in the rates of association and dissociation of the complexes (ka and kd, respectively). Our experimental results are consistent with the literature showing GRIN1 underexpression in schizophrenic disorders as well as an increased risk of cervical, bladder, and kidney cancers and lymphoma during ASCL3 underexpression. The effect of allele A of the –27G>A SNP (rs1195040887) in the NOS1 promoter is suggestive of an increased risk of ischemic damage to the brain in carriers. A comparison of experimental TBP–TATA affinity values (KD) of wild-type and minor alleles with predicted ones showed that the data correlate well (linear correlation coefficient r = 0.94, p <0.01).

## Abstract (ru)

Гены GRIN1, ASCL3 и NOS1 связаны с различными фенотипами нервно-психических расстройств. Эти гены делают вклад в развитие шизофрении, болезней Альцгеймера и Паркинсона, эпилепсии и др. и ассоциируются также с различными онкологическими заболеваниями. Например, повышенная экспрессия ASCL3 наблюдается при раке молочной железы, NOS1 – в клеточных линиях рака яичников. Ранее на основе наших и литературных данных мы получили результаты, свидетельствующие в пользу того, что SNP, нарушающие эритропоэз, с большой вероятностью могут быть связаны с когнитивными и нервно-психическими расстройствами у человека. В настоящей работе исследовано влияние выявленных с помощью SNP_TATA_Z-tester неаннотированных SNP ТАТА-боксов промоторов генов GRIN1, ASCL3 и NOS1, участвующих в нервно-психических расстройствах и онкологических заболеваниях, на взаимодействие ТАТА-связывающего белка (ТВР). Для изучения in vitro кинетических характеристик образования комплексов ТВР/ТАТА и аффинности с помощью метода задержки ДНК в геле использованы двуцепочечные олигодезоксирибонуклеотиды, идентичные ТАТА-содержащим участкам промоторов генов GRIN1, ASCL3 и NOS1 (референсным и минорным аллелям), и рекомбинантный ТВР человека. Показано, например, что аллель «A» rs1402667001 промотора гена GRIN1 повышает аффинность ТВР/ТАТА в 1.4 раза, а аллель «С» ТАТА-бокса промотора гена ASCL3 снижает аффинность в 1.4 раза; при этом время жизни комплексов в обоих случаях уменьшается примерно на 20 % за счет изменения скоростей образования и диссоциации комплексов (ka и kd соответственно). Наши экспериментальные результаты согласуются с литературными данными, показывающими низкую экспрессию гена GRIN1 при шизофренических расстройствах и повышенный риск возникновения рака шейки матки, мочевого пузыря, почек и лимфомы при пониженной экспрессии гена АSCL3. Влияние аллеля «А» SNP –27G>A (rs1195040887) промотора гена NOS1 гипотетически может свидетельствовать о повышенном риске возникновения ишемического повреждения мозга у носителей. Сравнение экспериментальных значений аффинности (KD) ТВР/ТАТА «диких» (WT) и минорных аллелей c прогнозируемыми показало, что данные хорошо коррелируют друг с другом: коэффициент линейной корреляции r = 0.94 ( p < 0.01).

## Introduction

Previously, using Web service SNP_TATA_Comparator (Ponomarenko M. et al., 2015) and in vitro experiments, we studied effects of SNPs in TATA boxes within core promoters of human genes to predict potential SNP markers, for example, markers of obesity (Arkova et al., 2015), autoimmune diseases (Ponomarenko M. et al., 2016), Alzheimer’s disease (Ponomarenko P. et al., 2017), aggressiveness (Chadaeva et al., 2016), circadian rhythm disorders (Ponomarenko P. et al., 2016), anomalies of female reproductive potential (Chadaeva et al., 2018), erythropoiesis disorders (Sharypova et al., 2018), and resistance to anticancer therapy (Turnaev et al., 2016). Then, on the basis of our results and literature data, we made findings suggesting that SNPs that disrupt erythropoiesis are likely to be associated with cognitive and neuropsychiatric disorders in humans (Ponomarenko M. et al., 2020).

The aim of the current work was to search for and to experimentally verify in vitro the effects of unannotated SNPs in TATA boxes within promoters of genes NOS1, GRIN1, and ASCL3 (which are involved in neuropsychiatric disorders and cancers) on the affinity and kinetic characteristics of TBP–TATA complexes. Identification of causal regulatory mechanisms of diseases is becoming a common practice, but experimental annotation of variants in target genes, especially in regulatory regions, is still a major bottleneck for the use of such genetic data in personalized medicine. Therefore, experimental quantitative methods for annotating SNPs in regulatory regions of specific genes remain important and relevant. This paper presents predictions of effects of unannotated SNPs in TATA boxes of genes GRIN1, ASCL3, and NOS1 on the thermodynamic and kinetic characteristics of TBP–TATA complexes as well as the results of their experimental verification in vitro

The GRIN1 gene, located in chromosomal region 9q34.3, codes for the GluN1 (NR1) subunit of the N-methyl-Daspartate receptor (NMDAR) and plays a key role in synaptic functions (Sin et al., 2002). The protein encoded by GRIN1 is a critical subunit of N-methyl-D-aspartate receptors, which are members of the superfamily of glutamate receptor channels. The latter are heteromeric protein complexes with multiple subunits arranged to form a ligand-gated ion channel. These subunits play a key part in synaptic plasticity, which is thought to underlie memory and learning

The first meta-analysis and convergence analysis (Forero, 2020) of available genome-wide expression studies regarding epileptogenesis in humans and model animals made it possible to identify several major candidate genes, including GRIN1. Animal models and postmortem studies on patients’ brains have shown that transcription and expression levels of the gene of the GluN1 protein in schizophrenia differ from those in controls (conditionally healthy volunteers), although the changes varied among different regions of the brain (Ding et al., 2017).

The achaete-scute complex-like (ASCL) gene family consists of five members, namely ASCL1, ASCL2, ASCL3, ASCL4, and ASCL5. The ASCL3 gene (SGN1) is located on chromosome 11, and its product was originally characterized as a transcription factor specifically localized to cells of salivary gland ducts (Park et al., 2017). Dysregulation of ASCL family genes has been reported to play a key role in psychiatric and neurological disorders (Hanahan, Weinberg, 2011). All ASCL genes encode the basic helix-loop-helix transcription factors that control nervous system development (Rugel-Stahl et al., 2012); thus, they are called proneural genes. Expression of ASCL family genes and the impact of their products on cells are not limited to the nervous system. For example, ASCL family members have been shown to be expressed in progenitor cells during muscle and intestinal-cell differentiation (Fox, 1998).

Using bioinformatic analyses, researchers have determined potential involvement of several ASCL family members in the initiation and progression of tumors in various types of cancer. ASCL3 is overexpressed in breast cancer (Hanahan, Weinberg, 2011) but is underexpressed (relative to normal controls) in kidney, cervical, and bladder cancers as well as lymphoma and melanoma. Analysis of different subtypes of kidney tumors has revealed that ASCL3 is downregulated in renal oncocytoma. As has already been mentioned, in lymphoma and cancers of the cervix, bladder, kidney, and epithelium, a decrease in the expression of ASCL3 has been documented (Hanahan, Weinberg, 2011), suggesting that this gene is a suitable research object not only in psychiatric and neurological disorders but also in cancers.

The NOS1 gene codes for the major isoform of nitric oxide synthase and is widely expressed in all tissues, and NOS1 produces approximately 90 % of nitric oxide in the central nervous system (Akyol et al., 2004). The gene is mapped to chromosomal region 12q24. Several studies indicate that NOS1 variants are associated with such disorders as Alzheimer’s disease (Mishizen-Eberz et al., 2004), schizophrenia (Shinkai et al., 2002; Saadat, 2010), and Parkinson’s disease (Hancock et al., 2008; Yu et al., 2018). Using reverse-transcription polymerase chain reaction (RT-PCR), some authors (Freudenberg et al., 2015) demonstrated that protein expression of NOS1 is constitutively high in ovarian cancer cell lines and that mRNA expression of NOS1 varies among such cell lines. The results of that study mean that NOS1 promotes malignant characteristics of ovarian cancer cells, including proliferation, invasion, and chemoresistance, thereby constituting a potential therapeutic target

## Materials and methods

DNA sequences. Unannotated SNPs (from the GRIN1 gene: rs1402667001, from ASCL3: rs1049743008:с, and from NOS1: rs1195040887) were retrieved from the dbSNP database (Sherry et al., 2001). Promoter sequences within the [–100; –1] region relative to a transcription start site were retrieved from the Eukaryotic Promotor Database (EPD) (Praz et al., 2002), and the presence of TATA boxes in these regions was determined in the same database

Analysis of DNA sequences in silico. DNA sequences of human genes GRIN1, ASCL3, and NOS1 between nucleotide positions –100 and –1 upstream of the transcription start site, which were taken from the reference genome, were analyzed using our Web service SNP_TATA_Z-tester, which is a modified version of SNP_TATA_Comparator (Ponomarenko M. et al., 2015).

Synthetic double-stranded oligodeoxyribonucleotides (ODNs). For experimental verification, we used 26 bp ODNs identical to the reference and minor alleles of genes GRIN1, ASCL3, and NOS1; the ODNs were synthesized and then purified by polyacrylamide gel electrophoresis by Biosan (Novosibirsk, Russia).

Sequences of these double-stranded ODNs – identical to the promoter regions of genes GRIN1, ASCL3, and NOS1 containing TATA-like elements – were as follows (reference [wild type; WT] alleles and minor alleles): GRIN1 (WT) – 5′-tggagggggACAAAGACAgggtggtg-3′ GRIN1 (–34g>a) – 5′-tggaggaggACAAAGACAgggtggtg-3′ ASCL3 (WT) – 5′-tcgaaaaaTAAAATAAAAtaaaacat-3′ ASCL3 (–45T>c) – 5′-tcgaaaaaTAAAAсAAAAtaaaacat-3′ NOS1 (WT) – 5′-tgtttcctGATAGAAAaaaaaaatgg-3′ NOS1 (–27G>a) – 5′-tgtttcctGATAaAAAaaaaaaatgg-3′

Labeling of the ODNs at 5′ ends with 32Р-γАТР. To prepare labeled double-stranded ODNs, both their strands were labeled with 32P-γATP (Biosan, Novosibirsk, Russia) by means of T4 polynucleotide kinase (SibEnzyme, Novosibirsk, Russia), annealed at 95 °C (at an equimolar ratio), and slowly cooled to room temperature. The annealed duplexes were purified and analyzed by electrophoresis in a nondenaturing 15 % polyacrylamide gel followed by autoradiography on a phosphorimager, Molecular Imager PharosFX Plus (Bio- Rad, Hercules, CA, USA). Unlabeled duplexes were prepared in the same way and used without further purification by polyacrylamide gel electrophoresis

Isolation and purification of recombinant TATA-binding (TBP) protein. We used recombinant human TBP expressed in Escherichia coli BL21(DE3) cells via the pAR3038- hTBP plasmid (a gift from Prof. B. Puhg, Center for Gene Regulation, Department of Biochemistry and Molecular Biology, The Pennsylvania State University, University Park, Pennsylvania, USA). Expression and purification of TBP were performed according to ref. (Pugh, 1995), except for the concentration of isopropyl β-D-1-thiogalactopyranoside (1.0 instead of 0.1 mM) and induction time (3 instead of 1.5 h).

Determination of kinetic parameters (rates of formation and dissociation of complexes, ka and kd) and equilibrium dissociation constants, KD, of TBP–ODN complexes. Association rate constants (ka) and dissociation rate constants (kd), which characterize the rates of formation and disintegration of complexes, were determined by measuring the kinetics of TBP binding to the TATA-containing doublestranded ODNs identical to either a WT TATA box (reference allele) or a TATA box carrying an SNP. The experiments were conducted at several concentrations of the 32P-labeled ODNs and a fixed concentration of TBP (0.4 nM unless stated otherwise). TBP–ODN binding experiments were performed at 25 °C in a buffer composed of 20 mM HEPES-KOH (pH 7.6), 5 mM MgCl2, 70 mM KCl, 1 mM dithiothreitol, 100 μg/ml bovine serum albumin, 0.01 % of NP-40, and 5 % of XYZ, as described in detail before (Drachkova et al., 2014). The electrophoretic mobility shift assay was carried out in a native 5 % polyacrylamide gel in Tris-glycine buffer (pH 8.3) for 40 min at 10 °C. The gels were dried, and Imaging Screen-K (Kodak) for Molecular Imager PharosFX Plus (Bio- Rad) was exposed to the gels. Each screen was scanned on the phosphorimager, and the autoradiographs were quantified in Quantity One v.4.5.0 software (Bio-Rad).

Statistical analysis. The comparison of the predicted and experimental values of TBP–TATA complexes’ affinity for the “normal” and minor alleles was performed using the Statistica software package (StatsoftTM, Tulsa, OK, USA).

## Results and discussion

Transcription, as a rule, is the gene expression stage most sensitive to internal signals and external signals entering the cell and is the main mechanism that controls gene expression. Here, we analyzed its initial stage, i. e., the interaction of TBP with a promoter: the process that triggers the assembly of the transcription complex

The Table presents the results of in vitro verification of our predictions (made by the SNP_TATA_Z-tester Web service) regarding the effect of substitutions in the TATA boxes of genes GRIN1, ASCL3, and NOS1 on TBP–TATA affinity. These data include experimental affinity (KD) values for WT and minor alleles and association and dissociation constants (ka and kd, respectively), reflecting the rates of formation and disintegration of TBP–TATA complexes

**Table 1.**
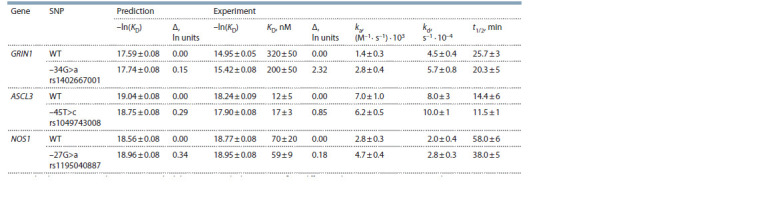
Experimental in vitro verification of our predictions of the effect of rs1402667001, rs1049743008, and rs1195040887 (from genes GRIN1, ASCL3, and NOS1, respectively) on affinity and kinetic characteristics of TBP–TATA complexes Note. The data are presented as mean ± standard deviation. KD = kd /ka; Δ, a TBP affinity difference (between ODNs containing and not containing an SNP) expressed in logarithmic units: Δ = –ln[KD, TATA(Mut)] – (–ln [KD, TATA]); t1/2 = ln2/kd.

**Fig. 1.**
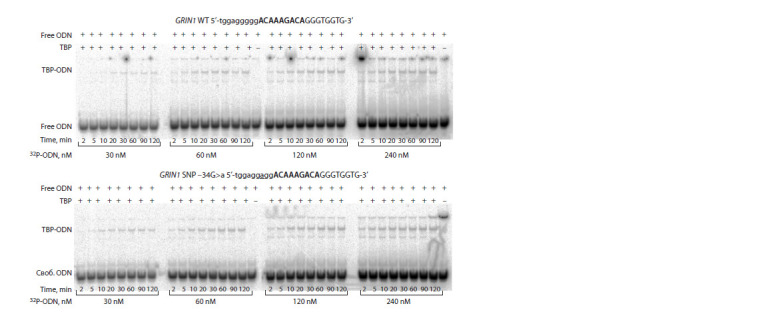
The electropherograms obtained to determine kinetic isotherms of TBP binding to the ODNs identical to the TATA-like element of the GRIN1 gene promoter: either the WT allele or minor allele “a” (SNP: –34G>a).

The results given in the Table were obtained by the electrophoretic mobility shift assay. Figure 1 shows electropherograms for the GRIN1 gene as an example.

**Fig. 2.**
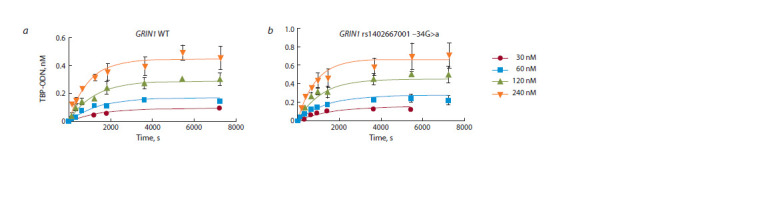
Kinetic isotherms of the TBP binding to the ODNs identical to the TATA box of the GRIN1 gene promoter, for either the WT allele (panel a) or minor allele “a” (panel b). The binding isotherms and ka and kd values were determined by means of the electropherograms (see Fig. 1) in GraphPad Prism 5 software.

As readers can see in the Table, the TBP–TATA affinity for WT GRIN1 can be described as low-specificity binding (KD = 320 nM). Although the TATA box sequence is AT-rich (ACAAAGACA), the third T, which has the greatest weight in the TATA box, is missing. In addition, conformational flexibility of the three As, which is clearly not enough to generate the conformation suitable for TBP binding, is blocked on both sides by high-melting-point base pairs containing C and G. The substitution (–34G>a) in the sequence flanking the TATA-like element did not significantly increase the flexibility of this DNA region, but the affinity strengthened almost 1.4-fold after introduction of the substitution (minor allele) as compared to the WT allele (KD = 200 nM). The rate of formation of the TBP–ODN complex increased twofold: 2.8 versus 1.4 M–1·s–1 (Fig. 2). The rate of complex dissociation was also slightly higher, by 20 %, in the case of the minor allele. Overall, these changes somewhat shortened the complex’s half-life (from 25 to 20 min), that is, made it less stable. Judging from the sequence of the ODN in question, which is identical to the region of the GRIN1 promoter, this promoter does not contain the TATA box consensus sequence but contains a G-rich box, which can bind to transcription factor SP3, which activates transcription of genes in chicken embryonic cortical neurons and represses it in undifferentiated cells (Chaudhary et al., 2017), as demonstrated in a study on the chicken GRIN1 gene.

The observed (in our study) weak affinity of TBP for the TATA-like element of the GRIN1 promoter, which implies gene underexpression (Liu et al., 2019), is in agreement with findings of low GRIN1 expression in animal models of schizophrenic disorders. On the basis of our data on enhanced TBP–TATA affinity for minor allele “a” (rs1402667001), we can theorize that carriers of this allele are at a reduced risk of schizophrenia as compared to carriers of WT allele G. It should be noted that despite active research, the role of GRIN1 in the etiology of schizophrenia remains uncertain. For instance, in ref. (Zhao et al., 2006), using Sanger DNA sequencing, researchers conducted a case-control study to investigate the association between GRIN1 and the risk of schizophrenia in a population of northern China. Distributions of both a genotype and allele of rs117783907 (–1945G/t) significantly differed between the case group and control group (p < 0.0083). In that article, genotype frequencies of rs138961287 and rs11146020 are statistically significantly different too (p < 0.05), indicating that rs138961287, rs117783907, and rs11146020 are associated with schizophrenia. In another association study conducted in a northern Chinese Han population, allele “c” of rs11146020 was reported to reduce the risk of schizophrenia (Begni et al., 2003), although Saadat (2010) found that this allele is a risk factor of schizophrenia in an Italian population. Furthermore, a meta-analysis (Zwicker et al., 2018) suggests that allele “c” of rs11146020 is associated with an increased risk of schizophrenia, i. e., the results are inconsistent among studies. It is likely that among different ethnic groups, the influence of environmental and genetic factors and their interactions may differ in their impact on the risk of mental disorders (Zwicker et al., 2018). The risk of psychosis increases with the accumulation of multiple variants carrying a genetic risk and with exposure to multiple adverse environmental factors (Gray et al., 2015).

Some authors (Ding et al., 2017) investigated the expression of a large group of genes in the brains of patients with major depressive disorder and controls (postmortem analysis). The results showed elevated expression of most of glutamatergic genes (e. g., GRIN1, GRIN2A–D, GRIA2–4, GRIK1 and -2, and GRM1) tested in the dorsolateral prefrontal cortex (mainly in women). Based on these findings, it can be assumed that rs1402667001 (studied by us), which enhances the affinity of TBP for the TATA-like element, may be a candidate SNP marker of an increased risk of schizophrenia. Despite conflicting results, some researchers (Zou et al., 2020) believe that the association of GRIN1 with schizophrenia and other psychotic disorders is undeniable and that subunit NR1 encoded by this gene may be a promising therapeutic target in schizophrenia

As readers can see in the sequence of the ASCL3 promoter region, which is identical to the region where a TATA box is usually located (positions –20 to –70 relative to the transcription start site), it is enriched in A nucleotides and contains a T. The latter has the greatest weight in the TATA box sequence (for the binding to TBP) and can take the third position in our case. Accordingly, we observed strong affinity of the TBP–TATA complex: KD = 12 nM. SNP –45T > C (rs1049743008), replacing T with high-melting-point C, led to a 1.4-fold weakening of the affinity (KD = 17 nM), although the rate of formation of the TBP–TATA complex increased slightly (12 %), while the rate of dissociation increased a little more: by 20 %. As a consequence, the complex’s half-life with the minor allele is also slightly shorter (11.5 versus 14.4 min), i. e., stability decreased. Because changes in the affinity of the TBP–TATA interaction correlate with alterations of gene expression (Mogno et al., 2010), it can be hypothesized that carriers of the C allele (with weakened TBP–TATA affinity and ASCL3 expression) are at a higher risk of a malignant tumor: lymphoma and cancers of the cervix, bladder, epithelium, and kidneys. This notion is confirmed by the results in ref. (Hanahan, Weinberg, 2011), where a database analysis revealed that out of 21 analyzed tumor types, five correlate with the ASCL3 expression that is diminished to various degrees

The NOS1 promoter contains a TATA-like element with sequence GATAGAAA, to which TBP binds with 70 nM affinity. When high-melting-point G was replaced in our study by A, the affinity strengthened, albeit slightly: by 14 % (KD = 59 ± 9 nM). In this context, the rate of TBP–TATA complex formation (ka) increased by a factor of 1.7, while the dissociation rate of the complex (kd) accelerated by a factor of 1.4, and the lifetime of the complex diminished ~1.4-fold.

Based on the results in ref. (Zou et al., 2020) indicating that NOS1 inhibitors can effectively reduce the severity of ischemic brain damage, it can be theorized that the A allele (SNP –27G>A, rs1195040887) – with enhanced TBP–TATA affinity and gene expression – may be a candidate marker of an elevated risk of the ischemic brain injury associated with cerebral palsy. The association of NOS1 with various diseases points to a pleiotropic role of NOS1 in many physiological processes and potentially to a pathogenesis that is shared among these diseases.

**Fig. 3.**
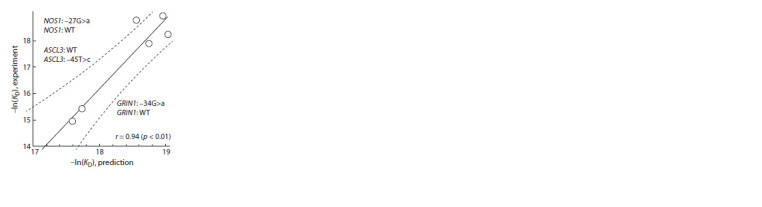
The significant correlation of the experimentally measured TBP–DNA affinity values with those predicted in silico by means of Web service SNP_TATA_Z-tester The dashed curves: a 95 % confidence interval for the regression line. The estimates were made using the Statistica package (StatsoftTM, USA).

Our comparison of the experimental affinity values (KD) of TBP–TATA complexes of reference (WT) alleles and minor alleles with those predicted by the SNP_TATA_Z-tester Web service (Ponomarenko M. et al., 2015) indicates that the data correlate well (linear correlation coefficient r = 0.94, p < 0.01) (Fig. 3).

Thus, we determined the affinity and kinetic characteristics of the interaction of TBP with TATA boxes containing unannotated SNPs. We found that these SNPs may be functionally significant and correlate with an increased risk of such neuropsychiatric diseases as schizophrenia and ischemic brain damage (associated with cerebral palsy) as well as the risk of malignant tumors: lymphoma and cancers of the cervix, epithelium, bladder, and kidneys.

## Conclusion

The results show effects of the analyzed SNPs (rs1402667001, rs1049743008, and rs1195040887) in the TATA boxes within promoters of genes GRIN1, ASCL3, and NOS1 on affinity and the rates of formation and disintegration of TBP–TATA complexes (ka and kd, respectively). Our experimental data suggest that the identified candidate SNP markers in neuronal genes can contribute to the development of not only neuropsychiatric but also oncological diseases, in agreement with the results obtained by other authors. Our findings about the influence of SNPs on TBP–TATA affinity and therefore on the expression of the genes in question point to their possible contribution to a higher risk of the diseases associated with these genes. Furthermore, our results have the potential to improve human health and to facilitate the development of new diagnostic markers

## Conflict of interest

The authors declare no conflict of interest.
